# Downregulation of LncRNA NORAD promotes Ox-LDL-induced vascular endothelial cell injury and atherosclerosis

**DOI:** 10.18632/aging.103034

**Published:** 2020-04-08

**Authors:** Weihua Bian, Xiaohong Jing, Zhiyu Yang, Zhen Shi, Ruiyao Chen, Aili Xu, Na Wang, Jing Jiang, Cheng Yang, Daolai Zhang, Lan Li, Haiyan Wang, Juan Wang, Yeying Sun, Chunxiang Zhang

**Affiliations:** 1 Department of Pharmacy, Binzhou Medical University, Yantai 264003, China; 2 Department of Gastroenterology, Henan Provincial People’s Hospital, People’s Hospital of Zhengzhou University, Zhengzhou 450003, China; 3 Department of Basic Medicine, Binzhou Medical University, Yantai 264003, China; 4 Children’s Heart Center, The Second Affiliated Hospital and Yuying Children’s Hospital, Wenzhou 325027, China

**Keywords:** NORAD, cell senescence, cell apoptosis, ox-LDL, IL-8

## Abstract

Long noncoding RNAs (lncRNAs) play important roles in the development of vascular diseases. However, the effect of lncRNA NORAD on atherosclerosis remains unknown. This study aimed to investigate the effect NORAD on endothelial cell injury and atherosclerosis. Ox-LDL-treated human umbilical vein endothelial cells (HUVECs) and high-fat-diet (HFD)-fed ApoE^−/−^ mice were used as *in vitro* and *in vivo* models. Results showed that NORAD-knockdown induced cell cycle arrest in G0/G1 phase, aggravated ox-LDL-induced cell viability reduction, cell apoptosis, and cell senescence along with the increased expression of Bax, P53, P21 and cleaved caspase-3 and the decreased expression of Bcl-2. The effect of NORAD on cell viability was further verified via NORAD-overexpression. NORAD- knockdown increased ox-LDL-induced reactive oxygen species, malondialdehyde, p-IKBα expression levels and NF-κB nuclear translocation. Proinflammatory molecules ICAM, VCAM, and IL-8 were also increased by NORAD- knockdown. Additionally, we identified the strong interaction of NORAD and IL-8 transcription repressor SFPQ in HUVECs. In ApoE^−/−^ mice, NORAD-knockdown increased the lipid disorder and atherosclerotic lesions. The results have suggested that lncRNA NORAD attenuates endothelial cell senescence, endothelial cell apoptosis, and atherosclerosis via NF-κB and p53–p21 signaling pathways and IL-8, in which NORAD-mediated effect on IL-8 might through the direct interaction with SFPQ.

## INTRODUCTION

Atherosclerosis is a multifactorial inflammatory process characterized by the deposition of oxidized low-density lipoprotein (ox-LDL), endothelial cell injury, and plaque accumulation in the vascular walls [1–3]. As a main risk factor of atherosclerosis, hypercholesterolemia triggers the deposition of oxidized low-density lipoprotein (ox-LDL) under the intima in vascular walls [1]. Ox-LDL induces reactive oxygen species (ROS) overproduction, adhesion molecule release, and endothelial cell injury through Low-density lipoprotein receptor 1 (LOX-1)-ROS-NF-κB and p53-Bax/Bcl-2-caspase-3 pathways [1, 4–6]. Endothelial cell injury and endothelial dysfunction is believed to be the initial step in an atherosclerosis process [3, 7].

Atherosclerosis is also an age-related disease prevalent in the elderly population [8]. A strong staining of the senescence marker senescence associated-beta-galactosidase (SA-β-gal) has been found in atherosclerotic lesions of coronary arteries and the senescence of vascular endothelial cells may play a key role in vascular aging and atherosclerosis development [9, 10]. Ox-LDL could induce endothelial cell senescence with excessive lipid deposition and senescence-associated protein overexpression [10]. Both p21 and p53 are found to be the key signaling molecules involved in endothelial cell senescence and the followed atherogenesis [11–13].

Long noncoding RNAs (lncRNAs) are RNAs with a length of more than 200 nucleotides [14]. They participate in cell proliferation, autophagy, apoptosis, and senescence [14, 15]. An abnormal lncRNA expression is associated with vascular cell dysfunction. LncRNAs also play crucial roles in regulating the functions of endothelial cells and vascular inflammation, suggesting the regulatory function of lncRNAs in atherosclerosis development [16].

NORAD is a newly characterized lncRNA activated by DNA damage and implicated in maintaining genomic stability and regular mitosis [17, 18]. It is abundantly expressed and highly conserved among mammals. In human genome, NORAD gene is composed of only one exon with 5.3 kb transcript in length and located at Chr20q11.23 [19]. NORAD transcripts are located both in the cytoplasm and nucleus [18, 20]. Forty-one proteins including the IL-8 transcriptional repressor SFPQ have been found to bind specifically to NORAD. Among the 41 proteins, 71% reside in the nucleus, and only 5% reside in the cytoplasm. Many of the 41 proteins play crucial roles in DNA replication and repair in the nucleus [20]. In nucleus, NORAD have been verified to interact with RBMX and be required to assemble a topoisomerase complex, namely, NORAD-activated ribonucleoprotein complex 1, which is involved in genome stability [20]. In the cytoplasm, NORAD binds to PUMILIO and modulates the mRNA levels of PUMILIO targets, which are rich in genes involved in cell proliferation and division [18]. In NORAD knockout mice, NORAD deletion leads to overactivity of PUMILIO, which inhibits genes critical to normal mitosis and leads to the accumulation of aneuploidy cells. NORAD deletion can also inhibit the genes that maintain mitochondrial homeostasis through the overactivation of PUMILIO, leading to significant mitochondrial dysfunction. In addition, NORAD knockout mice exhibit a multisystem degenerative phenotype similar to premature aging. To date, the roles of NORAD in diseases have been mainly focused on its effects on tumors. For example, NORAD could promote the development of ovarian cancer, gastric cancer, and pancreatic cancer colorectal cancer [21–24]. No studies have been reported on the effects of NORAD on endothelial cell injury and atherosclerosis.

Apolipoprotein E (ApoE) is a lipid carrier and can remove lipoproteins in arteries with an antiatherosclerosis activity [25]. ApoE-knockout mice fed with a high-fat diet (HFD) show features of atherosclerosis with high serum lipid levels and have been considered as an ideal model for studying atherosclerosis [26]. Therefore, in the current study, we used ApoE-knockout mice on a high-fat diet to establish an atherosclerosis model.

In this study, we aimed to investigate the effects of NORAD on endothelial cell injury, endothelial cell senescence and atherosclerosis and the potential molecular mechanisms involved in cultured human umbilical vein endothelial cells (HUVEC) *in vitro* and in ApoE-knockout mice *in vivo*.

## RESULTS

### NORAD-knockdown aggravates ox-LDL-induced reduction of cell viability and cell apoptosis in HUVECs

**Figure 1:**
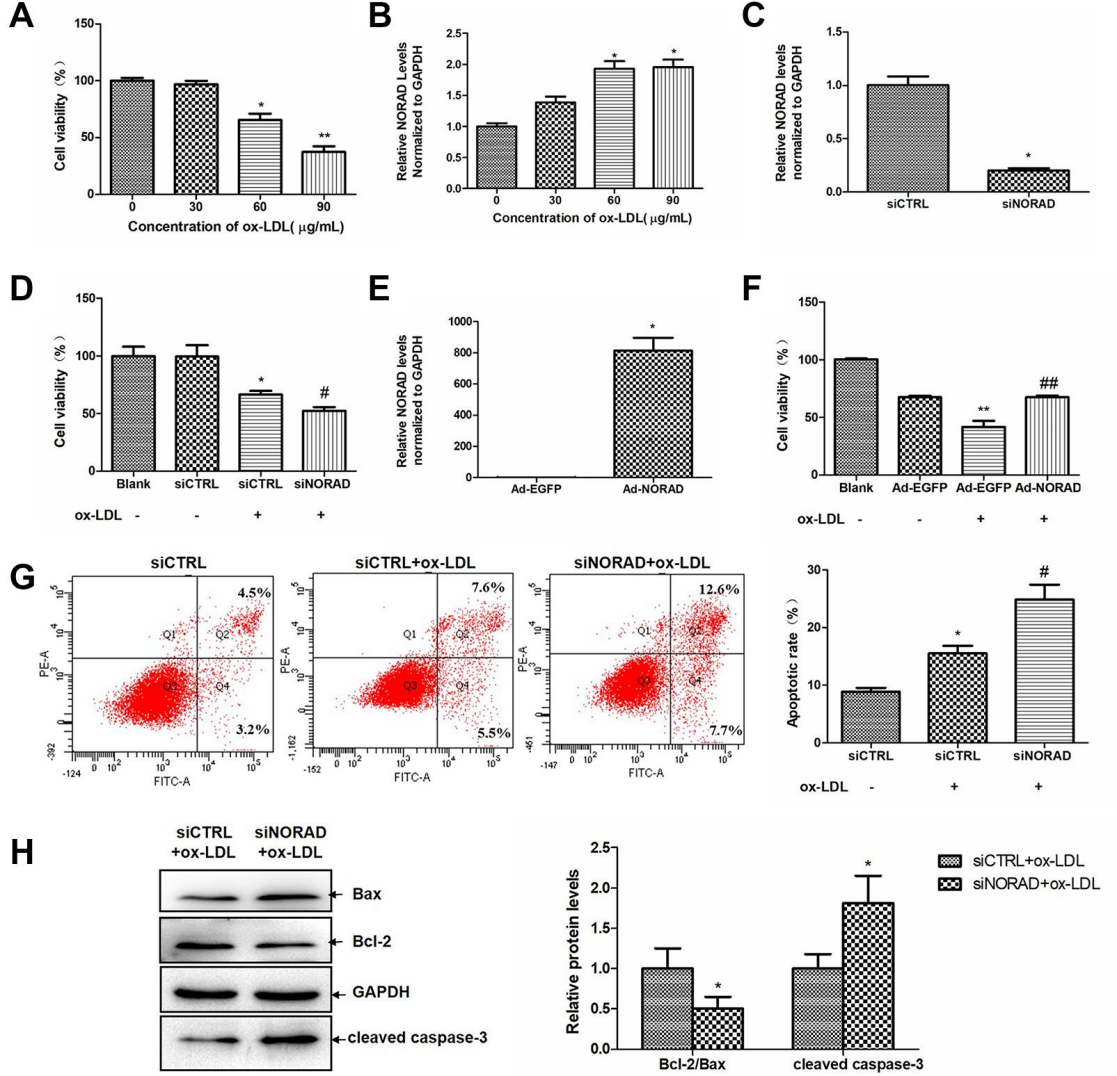
**NORAD-knockdown aggravates ox-LDL-induced viability reduction and apoptosis of HUVECs.** (**A**) Dose-dependent effect of ox-LDL on cell viability in HUVECs. Cell viability was measured after HUVECs were treated with 0-90 μg/mL of ox-LDL for 24 h by the CCK-8 assay. The data in each group were normalized with the group treated with 0 μg/mL ox-LDL. (n = 6, *P < 0.05 and ^##^P < 0.01 vs. group treated with 0 μg/mL ox-LDL, respectively). (**B**) Dose-dependent upregulation of NORAD expression in HUVECs treated with 0-90 μg/mL of ox-LDL for 24 h (n = 3, *P < 0.05 vs. group treatment with 0 μg/mL ox-LDL). (**C**) HUVECs were transfected with siNORAD or scrambled siCTRL. NORAD levels were analyzed through qRT-PCR (n = 3, ^*^P < 0.05 vs. siCTRL). (**D**) NORAD-knockdown suppressed the viability of ox-LDL-treated HUVECs. The effect of siNORAD on the cell viability was measured via a CCK-8 assay. Cells treated without both siRNA and ox-LDL were used as blank control. (n = 3, ^*^P < 0.05 vs. siCTRL, ^#^P < 0.05 vs. siCTRL+ox-LDL). The data of each group were normalized to the blank groups. (**E**) HUVECs were infected with Ad-NORAD or Ad-EGFP. NORAD levels were analyzed through qRT-PCR (n = 3, *P < 0.05 vs. Ad-EGFP). (**F**) The effect of NORAD overexpression on the cell viability was measured via a CCK-8 assay. Cells treated without both adenovirus and ox-LDL were used as blank control. (n = 6, **P < 0.01 vs. Ad-EGFP, ^##^P < 0.01 vs. Ad-EGFP+ox-LDL). The data of each group were normalized to the blank groups. (**G**) NORAD- knockdown increased ox-LDL-induced cell apoptosis. The apoptosis rate was detected through flow cytometry by using annexin V-FITC/PI double staining. The apoptotic rate was analyzed in terms of the percentage of the lower and upper right quadrants (n = 3, ^*^P < 0.05 vs. siCTRL, ^#^P < 0.05 vs. siCTRL+ox-LDL). (**H**) Western blot was used to analyze the expression levels of Bcl-2, Bax and cleaved caspase-3. The results were analyzed with Image J. Values were shown as mean ± SD (n = 3). ^*^P < 0.05 vs. siCTRL+ox-LDL.

Ox-LDL was used to mimic endothelial injury caused by lipid accumulation in atherosclerosis. Figure 1A showed a dose-dependent decline in the cell activity of HUVECS after exposure to ox-LDL (from 0 to 90 μg/mL). Figure 1B showed a dose-dependent increase in the NORAD expression level in HUVECS after exposure to ox-LDL (from 0 to 90 μg/mL). The dose of 60 μg/mL ox-LDL was selected for the subsequent experiments.

Small interfering RNA-targeting NORAD (siNORAD) was transfected into HUVECs to knockdown the NORAD expression. The NORAD knockdown was verified and assessed through reverse transcription- quantitative polymerase chain reaction (RT-qPCR). The NORAD expression in siNORAD cells was knocked down by 80% compared with that in control small-interfering RNA (siCTRL) cells (Figure 1C). HUVECs transfected with NORAD or control siRNA were treated with 60 μg/mL ox-LDL for 24h. HUVEC treated without both control siRNA and ox-LDL was named as the blank control group. The effect of NORAD-knockdown on the ox-LDL-induced reduction of HUVEC viability was detected with Cell Counting Kit-8 (CCK-8). Figure 1D showed that the cell viability in the siCTRL group with ox-LDL treatment was significantly decreased to 62.95% compared with the siCTRL cell without ox-LDL treatment (P < 0.05). The cell viability of siNORAD HUVECs with ox-LDL treatment was suppressed by 14.07% compared with that of siCTRL cells treated by ox-LDL. We also detect the cell viability in HUVECs with NORAD overexpression. HUVECs were infected with adenovirus Ad-NORAD or control Ad-EGFP and then treated with ox-LDL for 24h. As shown in Figure 1E, the NORAD expression was significantly increased in Ad-NORAD-infected cells. The cell viability of Ad-NORAD HUVECs with ox-LDL treatment was increased by 25.9% compared with that of Ad-EGFP cells treated by ox-LDL (Figure 1F).

The effect of NORAD-knockdown on the ox-LDL-induced apoptosis of HUVECs was detected via Annexin V–FITC/PI double staining. As shown in Figure 1G, NORAD knockdown intensively increased ox-LDL-induced cell apoptosis. The effect of NORAD-knockdown on apoptosis-related Bcl-2 family proteins and cleaved caspase-3 were assessed in ox-LDL-treated HUVECs through western blot analysis. As shown in Figure 1H, NORAD-knockdown significantly reduced the ratio of Bcl-2 to Bax and increased the cleaved caspase-3 level (P < 0.05).

### NORAD-knockdown induces cell cycle arrest in G0/G1 and aggravates ox-LDL-induced cell senescence of HUVECs

**Figure 2:**
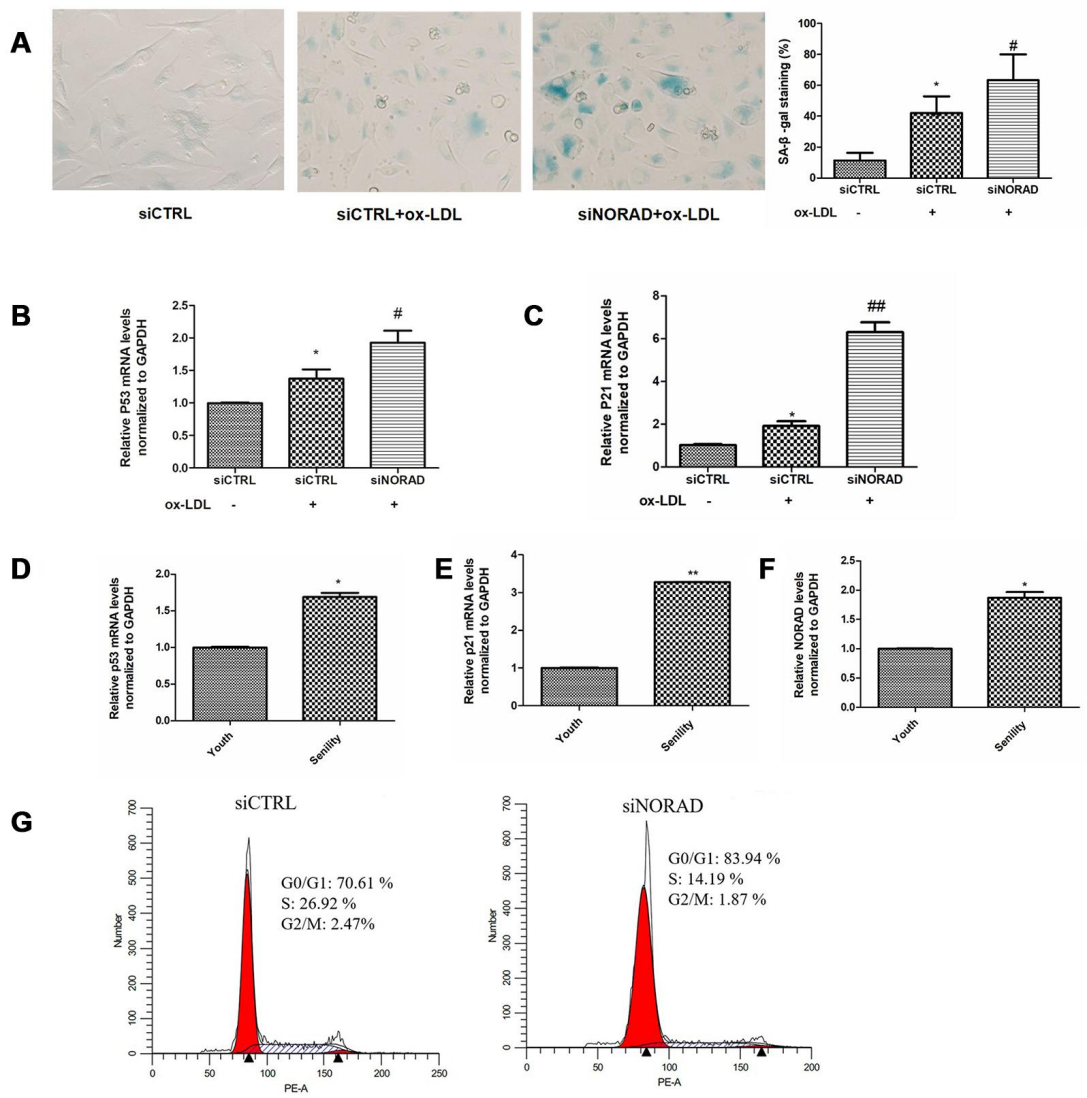
**NORAD-knockdown is results in the cell cycle arrest in G0/G1 phase and aggravates ox-LDL-induced senescence of HUVECs.** HUVECs were transfected with siNORAD or scrambled siCTRL. After transfection for 24 h, the cells were treated with 60 μg/mL ox-LDL for 24 h. (**A**) HUVECs were stained to determine SA-β-gal. NORAD-knockdown increased the number of positive-stained cells after they were treated with ox-LDL. Results were representative of three separate experiments. (**B**) mRNA expression of p53 in ox-LDL-treated HUVECs through RT-qPCR (n = 3, ^*^P < 0.05 vs. siCTRL, ^#^P < 0.05 vs. siCTRL+ox-LDL). (**C**) mRNA expression of p21 in ox-LDL-treated HUVECs through RT-qPCR (n = 3, ^*^P < 0.05 vs. siCTRL, ^##^P < 0.01 vs. siCTRL+ox-LDL). (**D**) mRNA expression of p53 in young and senile HUVECs through RT-qPCR (n = 3, ^*^P < 0.05 vs. Youth). (**E**) mRNA expression of p21 in young and senile HUVECs through RT-qPCR (n = 3, ^##^P < 0.05 vs. Youth). (**F**) NORAD levels in young and senile HUVECs through qRT-PCR (n = 3, ^*^P < 0.05 vs. Youth). (**G**) NORAD silencing induced ox-LDL-treated cell cycle arrest at G0/G1 phase. Flow cytometry was used to detect the cell cycle distribution of ox-LDL-treated HUVECs.

SA-β-gal staining was performed to assess cell senescence. The positively stained cells in ox-LDL-treated siCTRL group were obviously higher than those in siCTRL group without ox-LDL treatment, which indicated that the cell injury model was successful (42% vs 11%, P < 0.05, Figure 2A). In ox-LDL treatment cells, the percentage of SA-β-gal-positive cells in siNORAD group was obviously enhanced compared with that in siCTRL group (63% vs 42%, P < 0.05, Figure 2A). Correspondingly, the mRNA levels of the aging-related proteins p53 and p21 were significantly increased in NORAD knock-downed cells (Figs. 2B, 2C). The mRNA levels of p53, p21 and NORAD in young (passage 3) and aged (passage 10) HUVECs were also detected, respectively. As shown in Figs. 2D and 2E, the p53 and p21 expression levels were increased in the aged group compared with those in youth group, which further confirmed the effect NORAD-knockdown on cell senescence. However, the NORAD expression levels increased by 87% in the aged group compared with those in youth group (P < 0.05). Those findings implied that NORAD might protect against cell senescence of HUVECs.

Considering that NORAD-knockdown could increase the expression of p53 and p21, which are important cell cycle regulators, we examined the effect of NORAD-downregulation on cell cycle progression. In ox-LDL treatment cells, the percentage of HUVECs in the G0/G1 phase was obviously enhanced in siNORAD group compared with that in siCTRL group, whereas the percentage of S-phase cells was decreased significantly (Figure 2G). Overall, these results suggested that the downregulation of NORAD could increase the expression of p53 and p21, leading to cell cycle arrest at the G0/G1 phase and to the subsequent cell senescence and apoptosis.

### NORAD-knockdown aggravates ox-LDL-induced oxidative stress and p-IKBα expression level in HUVECs

**Figure 3:**
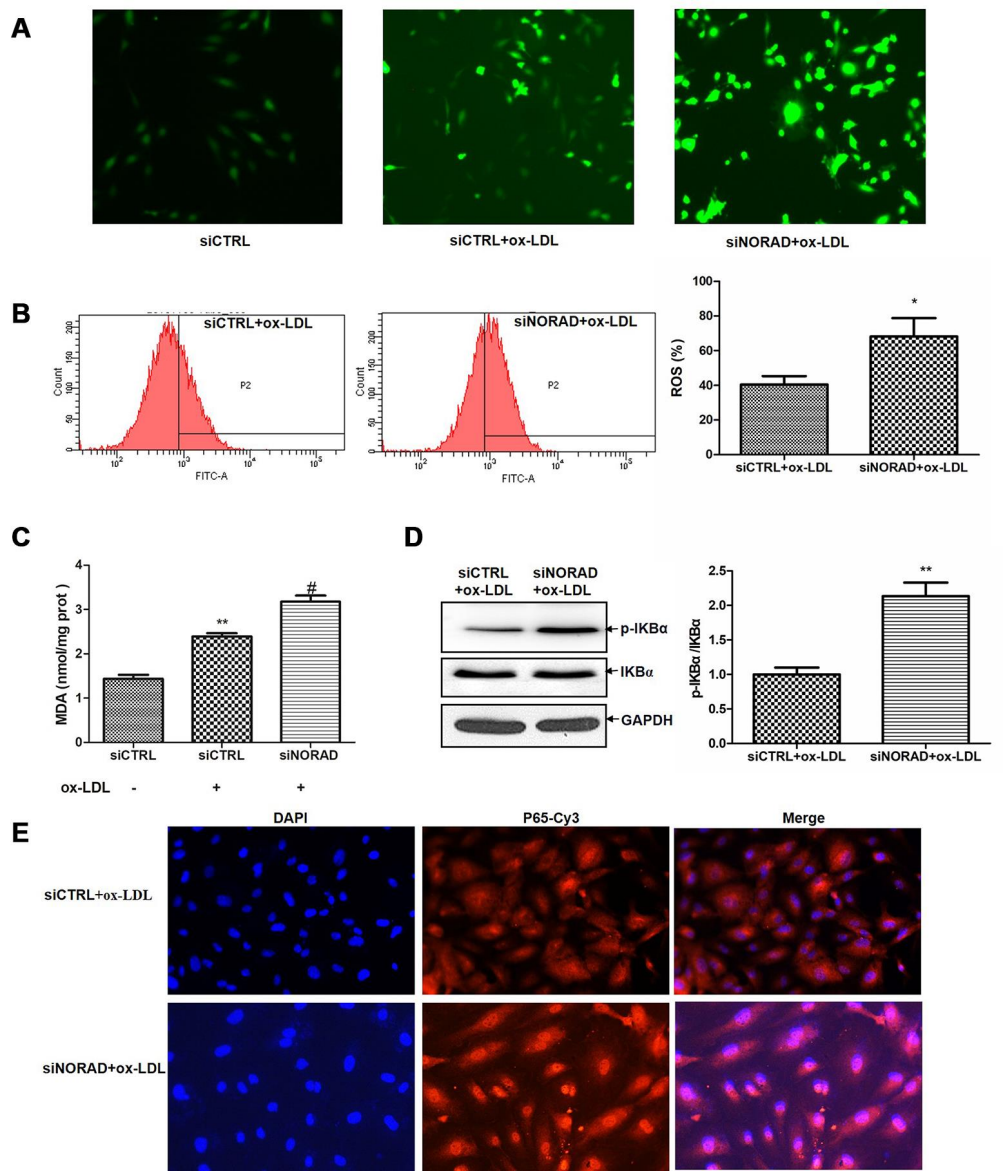
**NORAD-knockdown aggravates ox-LDL-induced oxidative stress and p-IKBα expression level in HUVECs.** HUVECs were transfected with siNORAD or scrambled siCTRL. After transfection for 24 h, the cells were treated with 60 μg/mL ox-LDL for 24 h. (**A**) NORAD knockdown aggravated ox-LDL-induced ROS production. HUVECs were treated with 60 μg/mL ox-LDL for 3 h and incubated with DCF-DA for 25 min. The ROS levels were observed under an inverted fluorescence microscope. Images were representative of three separate experiments (n = 3). (**B**) Flow cytometry was used to detect the intracellular ROS levels. Data were from three separate experiments and described as mean ± SD. ^*^P < 0.05 vs. the siCTRL group. (**C**) MDA content in HUVECs treated with 60 μg/mL ox-LDL for 24 h. Data were shown as mean ± SD of three separate experiments. ^**^P < 0.01 vs. siCTRL, ^#^P < 0.05 vs. siCTRL+ox-LDL. (**D**) NORAD-knockdown increased the p-IKBα expression observed through western blot. The ratio of p-IKBα to IKBα was analyzed with ImageJ. Values were shown as mean ± SD (n = 3). ^**^P < 0.01 vs. siCTRL. (**E**) NORAD-knockdown increased NF-κB nuclear translocation by immunofluorescence. p65 was stained red with Cy3. Nuclei were stained blue with DAPI. Co-localization of p65 with nucleus is shown in purple.

ROS production is a signal to trigger cell senescence and apoptosis. Evidence suggests that ox-LDL can increase ROS production in HUVECs [4, 5], and the increased ROS is the main cause of oxidative stress. After ox-LDL treatment, the ROS level in siCTRL group was significantly increased. The ox-LDL-induced ROS level in siNORAD group was increased by NORAD-knockdown compared with that in siCTRL group (Figure 3A). We also determined the effect of NORAD-knockdown on ox-LDL-induced ROS production via flow cytometry. NORAD-knockdown increased the ROS level by 28% (P < 0.05; Figure 3B). As the increase in ROS could cause lipid peroxidation and lead to malondialdehyde (MDA) production, we thus determined the effect of NORAD-knockdown on ox-LDL-induced MDA production. The MDA level in ox-LDL-treated siCTRL group was increased compared with that in siCTRL group without ox-LDL treatment (P < 0.01; Figure 3C). The ox-LDL-induced MDA level in siNORAD group was increased by NORAD-knockdown compared with that in siCTRL group (P < 0.05; Figure 3C).

The increase in ROS can activate the NF-κB signaling pathway. NF-κB is a common transcription factor. The most common subunits of NF-κB is p65. When NF-κB is not activated, it forms a complex with IKBα and is distributed in the cytoplasm. In the presence of stimuli such as inflammatory factors, growth factors, or chemokines, IKBα is phosphorylated and subsequently degraded by the ubiquitin - proteasome pathway. When NF-κB and IKBα are depolymerized, NF-κB is activated and transported to the nucleus to facilitate NF-κB dependent gene transcription. So activation of NF-κB can be determined by p-IKBα level or by immunostaining to detect whether the main subunit of NF-κB, p65, has been transferred to the nucleus.

We determined whether NORAD-knockdown could regulate the ox-LDL-induced p-IKBα level and NF-κB nuclear translocation. As shown in Figure 3D, NORAD-knockdown induced a significant increase in the p-IKBα/IKBα level compared with that in siCTRL group (P < 0.05). In Figure 3E, The p65 protein emitted red fluorescence and the nuclei emitted blue fluorescence after stained with DAPI. The red and blue images were merged to produce images with purple fluorescence. Compared with siCTRL group, the nuclei of siNORAD group showed more purple color after ox-LDL treatment, indicating that obvious NF-κB nuclear translocation occurred in siNORAD group.

### NORAD knockdown increases the levels of ox-LDL-induced proinflammatory molecules in HUVECs

**Figure 4:**
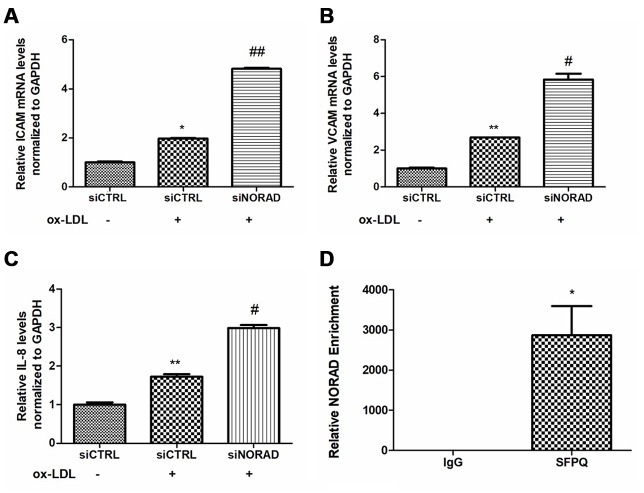
**Effects of NORAD-knockdown on ox-LDL-induced proinflammatory molecules in HUVECs.** (**A**) mRNA expression of ICAM-1 in ox-LDL-treated HUVECs observed through qRT-PCR (n = 3, ^*^P < 0.05 vs. siCTRL, ^##^P < 0.01 vs. siCTRL+ox-LDL). (**B**) mRNA expression of VCAM in ox-LDL-treated HUVECs observed through qRT-PCR (n = 3, ^**^P < 0.01 vs. siCTRL, ^#^P < 0.05 vs. siCTRL+ox-LDL). (**C**) mRNA expression of IL-8 mRNA in ox-LDL-treated HUVECs observed through qRT-PCR (n = 3, ^**^P < 0.01 vs. siCTRL, ^#^P < 0.05 vs. siCTRL+ox-LDL). (**D**) RIP assay confirmed the interaction of NORAD and SFPQ. HUVEC lysates were incubated with the anti-SFPQ or anti-IgG antibody, and the precipitated complexes were analyzed with qRT-PCR to investigate the expression of NORAD (n = 3, ^*^P < 0.05 vs. IgG).

We tested the levels of proinflammatory molecules, including ICAM, VCAM, and IL-8. After incubation with ox-LDL for 24 hours, the mRNA levels of ICAM, VCAM and IL-8 were significantly increased. The ox-LDL-induced ICAM, VCAM, and IL-8 levels in the siNORAD group were increased by NORAD-knockdown compared with those in siCTRL group (Figure 4A–4C).

Recently, SFPQ has been identified as one of NORAD-interacting proteins in HCT116 colon carcinoma cells [20]. SFPQ is a repressor of IL-8 transcription when it binds to the IL-8 promoter in HeLa TO cells [27]. To verify the potential interaction of NORAD and SFPQ in HUVECs, RNA-binding protein immunoprecipitation (RIP) assay was used. The NORAD enrichment by SFPQ was 2872-fold higher than that by the negative control IgG (P < 0.01, Figure 4D). The result suggested that NORAD was strongly coimmunoprecipitated with SFPQ in immunoprecipitation complexes from HUVECs. Thus, the IL-8 transcription repressor SFPQ might be a direct target gene in HUVECs, although additional experiments need to be performed to confirm it.

### NORAD-knockdown increases the serum lipid levels in ApoE^−/−^ mice

**Figure 5:**
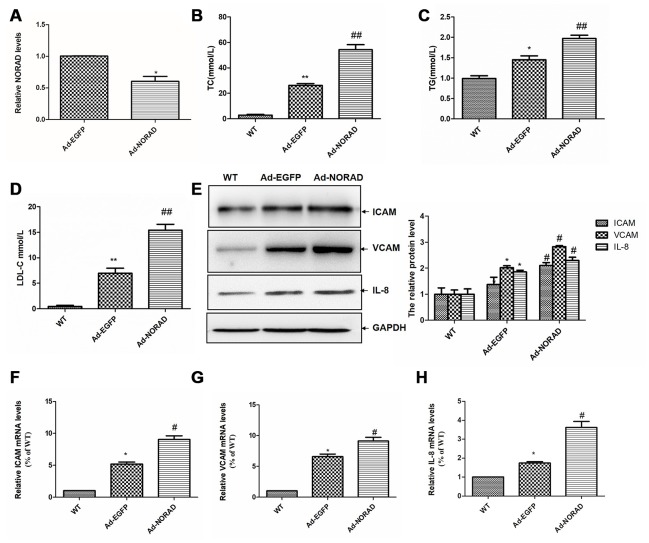
**NORAD-knockdown aggravates the abnormal serum lipid condition and increased the proinflammatory molecule expression induced by HFD in ApoE^−/−^ mice.** ApoE^−/−^ mice were given Ad-NORAD or control Ad-EGFP injection after 8 weeks of HFD, and HFD was maintained for another 8 weeks. C57BL/6 J mice fed with the normal diet were set as the WT control group. (**A**) NORAD levels in the aortic sinus were analyzed through qRT-PCR (n = 3, ^*^P < 0.05 vs. Ad-EGFP). Serum levels of lipids, TC (**B**), TG (**C**), and LDL-C (**D**) were detected. Values were shown as mean ± SD (n = 8). ^*^P < 0.05, ^**^P < 0.01 vs. WT group, and ^##^P < 0.01 vs. Ad-EGFP group. (**E**) Western blot was used to detect the ICAM, VCAM, and IL-8 expression levels (n = 8, ^*^P < 0.05 vs. WT group, and ^#^P < 0.05 vs. Ad-EGFP group). The mRNA levels of ICAM (**F**), VCAM (**G**), and IL-8 (**H**) in thoracic aortas were detected through qRT-PCR (n = 8, ^*^P < 0.05 vs. WT group, and ^#^P < 0.05 vs. Ad-EGFP group).

The ApoE^−/−^ mice were given Ad-NORAD or control Ad-EGFP injection after 8 weeks of HFD, and HFD was maintained for another 8 weeks to study the effect of NORAD knockdown on atherosclerosis development. The C57BL/6 J mice fed with normal diet were used as the wild type (WT) control group. To detect the adenovirus transduction efficiency, NORAD expression in thoracic aortas were examined at 3 days after adenovirus injection. The result showed the NORAD expression in Ad-NORAD group was knocked down by 40% compared with that in Ad-EGFP group (P < 0.05, Figure5A). The serum lipid levels were measured. The total cholesterol (TC) level was 2.92 ± 1.31 mmol/L in WT group, which was increased to 26.15 ± 3.64 mmol/L in Ad-EGFP group (P < 0.01, compared with the WT group) and to 54.33 ± 9.56 mmol/L in Ad-NORAD group (P < 0.01, compared with the Ad-EGFP group; Figure 5B). The triglyceride (TG) level was 0.99 ± 0.2 mmol/L in WT group, which was increased to 1.45 ± 0.28 mmol/L in Ad-EGFP group (P < 0.05, compared with the WT group), and further increased to 1.98 ± 0.23 mmol/L in Ad-NORAD group (P < 0.01, compared with the Ad-EGFP group; Figure 5C). The level of low-density lipoprotein cholesterol (LDL-C) was 0.45 ± 0.22 mmol/L in WT group, which was increased to 6.98 ± 0.96 mmol/L in Ad-EGFP group (P < 0.01, compared with the WT group), and further increased to 15.4 ± 1.16 mmol/L in Ad-NORAD groups (P < 0.01, compared with the Ad-EGFP group; Figure 5D). These results indicated that HFD increased the serum levels of TC, TG, and LDL-C levels in ApoE^−/−^ mice, and the increase was aggravated by NORAD-knockdown.

### NORAD-knockdown increases the levels of ICAM, VCAM, and IL-8 in the thoracic aortas of ApoE^−/−^ mice

We detected the effects of NORAD-knockdown on the mRNA and protein levels of ICAM, VCAM, and IL-8 in the thoracic aortas. Western blot results showed that the levels of VCAM, ICAM, and IL-8 levels in Ad-EGFP group were significantly higher than those in WT group. The expression levels of them in Ad-NORAD group were further increased compared with those in Ad-EGFP group (Figure 5E). The results were confirmed by qRT-PCR at the mRNA level (Figure 5F–5H).

### NORAD knockdown promotes atherosclerosis development in ApoE^−/−^ mice

**Figure 6:**
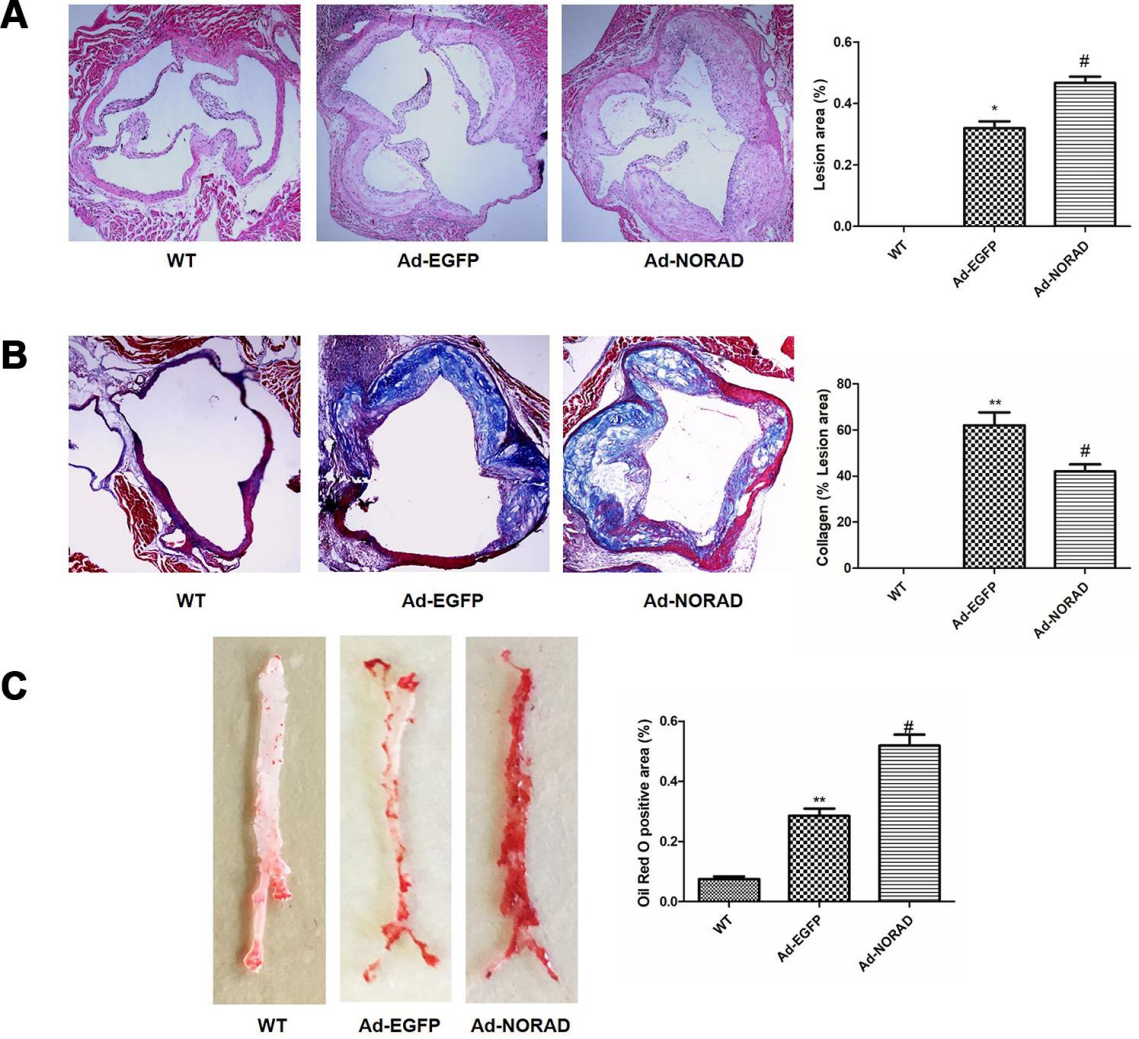
**NORAD-knockdown promotes atherosclerosis development in ApoE^−/−^ mice.** ApoE^−/−^ mice were given Ad-NORAD or control Ad-EGFP injection after 8 weeks of HFD, and the HFD was maintained for another 8 weeks. C57BL/6 J mice fed with the normal diet were used as the blank WT control group. (**A**) Representative micrographs and quantification of lesion area in the aortic sinus observed through H&E staining. (**B**) Representative micrographs and quantification of the collagen fibers area in the aortic sinus observed through Masson staining. (**C**) Representative en face images of the entire aorta with Oil Red O staining. The atherosclerotic lesion areas were stained red. Oil Red O-positive areas were analyzed with ImageJ. Data were shown as mean ± SD, n = 8. ^**^P < 0.01 vs. WT group, ^*^P < 0.05 vs. WT group, and ^#^P < 0.05 vs. Ad-EGFP group.

Hematoxylin and eosin (H&E), Masson and Oil Red O staining were used to evaluate the effects of NORAD-knockdown on atherosclerosis in ApoE^−/−^ mice. The H&E staining results showed that the aortic sinus plaque area was significantly increased in Ad-NORAD group compared with that in Ad-EGFP group (Figure 6A). The Masson staining results showed that the collagen content in aortic sinus plaque was decreased in Ad-NORAD group compared with that in Ad-EGFP group, suggesting that downregulation of NORAD might decrease the stability of atherosclerotic plaque (Figure 6B). The Oil Red O staining showed that the lipid deposition area in the atherosclerotic plaque was significantly increased in Ad-NORAD group compared with that in Ad-EGFP group (Figure 6C).

## DISCUSSION

This study has demonstrated how NORAD could affect the injury responses of endothelial cells and atherosclerosis in ApoE^−/−^ mice via siNORAD transfection *in vitro* and adenovirus Ad-NORAD injection *in vivo*. Several discoveries have been identified in this research project. First, NORAD- knockdown could aggravates ox-LDL-induced oxidative damage and subsequent inflammation, leading to cell senescence and apoptosis in HUVECs. Second, NORAD-knockdown could increase the serum lipid levels and enhance the atherosclerotic lesions in ApoE^−/−^ mice fed with HFD. In addition, we have verified that NORAD has strong interaction with the IL-8 transcription repressor SFPQ, which might be molecular mechanism in NORAD-mediated effect on the expression of IL-8. The role of SFPQ in NORAD-mediated effects on endothelial cells should be determined in future studies.

Many lncRNAs are closely related to the function of vascular endothelial cells. For example, lncRNA-RP11-714G18.1 inhibits vascular cell migration by targeting LRP2BP [28]. Let-7e regulates the NF-κB pathway of vascular endothelial cells through competing endogenous RNA crosstalk [29]. NORAD is a novel lncRNA with high conservatism from mice to humans and is abundant in many cells and tissues including endothelial cells. NORAD may have effects on cell proliferation, invasion, and apoptosis in cancer cells and is related to the development of cancers [19]. The mechanisms of NORAD in cancers are complex, which include many factors and signaling pathways. For example, NORAD silencing reduces ovarian cancer cell activity, migration, and invasion but induces cell apoptosis through the NORAD/miR-608/STAT3 axis [21]. NORAD promotes gastric cancer cell proliferation and migration by regulating the miR-608/FOXO6 pathway [22]. NORAD increases epithelial–mesenchymal transformation and promotes pancreatic cancer metastasis via the NORAD/hsa-miR-125a-3p/RhoA axis [23]. NORAD knockdown also induces colorectal cancer cell apoptosis through the NORAD/miR-202-5p axis [24]. However, the effect of NORAD on vascular endothelial cell injuries and atherosclerosis remains unclear.

ox-LDL-mediated endothelium apoptosis is an important cause of atherosclerosis [30]. The damage of ox-LDL to the vascular wall is manifested in the promotion of lipid deposition, occurrence of inflammatory reaction, release of metalloproteinases, and apoptosis of endothelial cells, macrophages, and vascular smooth muscle cells. A large amount of ox-LDL accumulation has been found in an injured area of the arterial wall, and in vascular endothelial cells undergo apoptosis. Bax, Bcl-2, and caspase-3 expression is abnormal in ox-LDL-induced HUVECs, which is related to ox-LDL-induced injury responses of the endothelial cells [31]. In our study, we have found that NORAD-knockdown decreases the cell viability and increases ox-LDL-mediated HUVEC apoptosis with the decreased ratio of Bcl-2 to Bax and the increased expression of cleaved caspase 3. The results indicated that NORAD may have a protective role against ox-LDL-mediated HUVEC apoptosis via Bax, Bcl-2, and caspase-3. Oxidative stress is one of the main factors that induce apoptosis and characterized by excessive ROS production [32]. A previous study showed that Ox-LDL could bind to its receptor LOX-1 and induce HUVEC apoptosis through LOX-1-ROS-NF-κB signaling cascade [4]. In our study, the ROS production and NF-κB activation were measured to detect whether these signal transduction pathways were involved in the effects of NORAD-knockdown. We also detected the level of MDA, which is one of the oxidative products of ROS. The results have demonstrated that NORAD-knockdown could indeed aggravate ox-LDL-induced oxidative stress and p-IKBα/IKBα expression level in HUVECs.

In addition to apoptosis, vascular endothelial cell senescence occurs in atherosclerotic arteries and may be an important initial cellular change in atherosclerosis progression [33]. Vascular endothelial cell senescence is detected with two main methods: β-galactosidase staining and cell cycle assay. Oxidative stress is a well-known factor that could induce cell senescence [34]. In addition, the p53–p21 axis triggers cell senescence in response to ROS [35]. p21, as a cyclin-dependent kinase inhibitor, can induce cell cycle arrest at the G0/G1 phase [36]. In the present study, NORAD-knockdown enhanced the SA-β-gal activity and the p53 and p21 expression in ox-LDL-treated HUVECs, which demonstrated that NORA- knockdown aggravated the ox-LDL-induced cell senescence of HUVECs via the p53–p21 pathway. Kopp et al. have reported that the NORAD-deficient mice show aging signs with the thinner and grayer fur, abnormally curved spines and shorter lifespan. NORAD-deficient mice also show cellular senescence with abnormal number of chromosomes and mitochondrial defects [37]. Our findings have provided direct evidence that NORAD deficiency leads to cell senescence in HUVECs. Cell senescence can be induced by cell cycle arrest [35]. Our results showed that NORAD-knockdown resulted in cell cycle arrest in G0/G1phase. Our results indicate that NORAD-knockdown induces cell cycle arrest, senescence, and apoptosis in ox-LDL-treated HUVECs via the NF-κB and p53-p21 pathways.

After vascular endothelial cell injury, cells secrete chemokines, ICAM, VCAM, and other active substances, which stimulate white blood cells to the injured sites. Monocytes migrate to the subendothelial space and become the activated macrophages, which uptake ox-LDL to become foam cells, and the atherosclerotic plaque is thus formed [38]. The expression of the endothelial adhesion molecules ICAM and VCAM is controlled by NF-κB [39]. Considering that the NF-κB pathway is activated by silencing NORAD, we further examined the changes of ICAM and VCAM levels. We demonstrated that NORAD-knockdown increased the VCAM and ICAM levels in ox-LDL-induced HUVECs. IL-8 is another widely studied gene that is relevant to atherogenesis. In atherosclerosis, ox-LDL induces the overexpression of IL-8 by the vascular endothelial cells, which could activate macrophages and aggravate inflammatory responses [40]. Our study showed that NORAD-knockdown increased the IL-8 levels in ox-LDL-treated HUVECs. A recent study reported that SFPQ binds to the promoter region of IL-8 DNA to inhibit IL-8 expression. In the current study, the RIP results showed that NORAD interacted tightly with SFPQ, which is consistent with previous screening results via a RAP technique. LncRNAs may act as a scaffold molecule that recruits proteins to form complexes, which play important roles in chromatin remodeling and then enhance or suppress inflammatory transcription [41, 42]. For example, as a scaffold molecule, lincRNA–EPS interacts with heterogeneous nuclear ribonucleoprotein L and forms a complex that regulates gene transcription by binding to the regulatory regions of immune response genes [42]. It might be possible for NORAD to act as a scaffold molecule and bind to SFPQ to form a complex, which inhibited IL-8 transcription. However, the above proposed mechanism should be further confirmed via other approaches in further studies.

ApoE^−/−^ mice are a classical animal model to develop hypercholesterolemia and atherosclerosis [43]. By using this model, we found that NORAD knockdown significantly increased the serum levels of total TC, TG, and LDL-C levels, which are among the characteristics of lipid disorders in atherosclerosis. ICAM, VCAM, and IL-8 were remarkably increased in ApoE^−/−^ mice with NORAD-knockdown. NORAD knockdown also significantly increased the aortic plaque area and lipid deposition, and decreased the stability of atherosclerotic plaque. These findings suggest that NORAD-knockdown promotes atherosclerosis development in ApoE^−/−^ mice.

In conclusion, our experiments have suggested that lncRNA NORAD attenuates endothelial cell senescence, endothelial cell apoptosis, and atherosclerosis via NF-κB and p53–p21 signaling pathways and IL-8, in which NORAD-mediated effect on IL-8 might through the direct interaction with its transcription repressor SFPQ.

## MATERIALS AND METHODS

### Animals and treatment

Six-week-old male ApoE^−/−^ mice and age-matched male C57BL/6 J mice were purchased from GemPharmatech Co., Ltd. (Nanjing, China). The mice were exposed to standard light conditions at 25 °C. ApoE^−/−^ mice were given HFD from the age of 8 weeks. HFD (21% fat, 0.25% cholesterol) was purchased from Beijing Keao Xieli Feed Company, Ltd. After the ApoE^−/−^ mice were fed with HFD for 8 weeks, they were given adenovirus Ad-NORAD or control Ad-EGFP at a dosage of 2×10^9^ pfu/mouse via tail vein injection and continued on HFD for another 8 weeks until they were 24-week-old. Adenovirus Ad-NORAD and control Ad-EGFP were purchased from Genechem Co., Ltd. (Shanghai, China). The C57BL/6 J mice fed with a normal diet were labeled as the WT control group.

### Cell culture, RNA interference and Adenovirus infection

Primary HUVECs were obtained from Shanghai AllCells Biotech Co., Ltd. (Shanghai, China) and cultured in endothelial cell culture medium (AllCells Biotech Co., Ltd., Shanghai, China). The cells were cultured in a humidification atmosphere of 5% CO_2_. Cells of passages 3–5 were used in this study, except for senescent cells which were from passage 10.

For RNA interference assay, siNORAD and negative siCTRL were from GenePharma Co., Ltd. (Shanghai, China). The siNORAD sequence was composed of the sense strand GCUGUCGGAAGAGAGAAAUTT and the antisense strand AUUUCUCUCUUCCGACAGCTT. The sequence of siCTRL consisted of the sense strand UUCUCCGAACGUGUCACGUTT and the antisense strand ACGUGACACGUUCGGAGAATT. HUVECs were transfected with siRNAs by using RNAIMAX (Invitrogen, CA, USA). For ox-LDL treatment, the cells were exposed to 60 μg/mL of ox-LDL after transfection for 24 h. For adenovirus infection assay, HUVECs were infected with adenovirus Ad-NORAD (Genechem, Shanghai, China) to overexpress NORAD. Adenovirus Ad- EGFP was used as the control.

### Reverse transcription- quantitative polymerase chain reaction (RT-qPCR)

The HUVECs in each group were treated and suspended in Trizol reagent (Invitrogen). Total RNA was extracted and converted into cDNA by using a PrimeScript^TM^ RT reagent kit with a gDNA eraser (Takara, Otsu, Japan). SYBR Premix Ex Taq II (Takara, Otsu, Japan) was then used to amplify CDNA through qPCR. The sequences of oligonucleotide primers used for qPCR were listed in Table 1. The 2^−ΔΔCT^ method was used to analyze the relative fold change.

**Table 1:** The sequences of primers used for RT-qPCR.

**Gene Name**	**Primer Sequence (Forward)**	**Primer Sequence (Reverse)**
NORAD (human)	TGATAGGATACATCTTGGACATGGA	AACCTAATGAACAAGTCCTGACATACA
p53 (human)	CCTCCTCAGCATCTTATCCG	CAACCTCAGGCGGCTCATAG
p21 (human)	ACCGAGACACCACTGGAGGG	CCTGCCTCCTCCCAACTCATC
GAPDH (human)	GGAGCGAGATCCCTCCAAAAT	GGCTGTTGTCATACTTCTCATGG
IL-8 (human)	CTTGGCAGCCTTCCTGATTT	ACAACCCTCTGCACCCAGTT
ICAM-1 (human)	ATGCCCAGACATCTGTGTCC	GGGGTCTCTATGCCCAACAA
VCAM-1 (human)	CTTAAAATGCCTGGGAAGATGGT	GTCAATGAGACGGAGTCACCAAT
GAPDH (mouse)	AGGTCGGTGTGAACGGATTTG	TGTAGACCATGTAGTTGAGGTCA
IL-8 (mouse)	TCGAGACCATTTACTGCAACAG	CATTGCCGGTGGAAATTCCTT
ICAM (mouse)	TCACCAGGAATGTGTACCTGACA	ATCACGAGGCCCACAATGAC
VCAM (mouse)	TCATCCCCACCATTGAAGAT	TGAGCAGGTCAGGTTCACAG

### Cell viability assay

HUVECs were seeded on a 96-well plate and cultured for 24 h before siRNA transfection. After transfection for 24 h, the HUVECs were treated with ox-LDL (60 μg/mL) for 24 h. For NORAD overexpression, after infection with Ad-EGFP or Ad-NORAD for three days, the HUVECs were treated with ox-LDL (60 μg/mL) for 24 h. Cell viability was then measured via a CCK-8 assay. CCK-8 solution (10 μL, Solarbio Biotechnology, Beijing, China) was added to 90 μL of culture medium, and the plates were incubated at 37 °C for 2 h. A microplate reader (Tecan) was used to measure the absorbance at 450 nm. The results are shown as percentages of the values from the blank group without ox-LDL treatment.

### Cell apoptosis assay

HUVECs were seeded on six-well plates prior to transfection at a cell density of 1.5 × 10^5^ cells/well. After transfection for 24 h, the cells were treated with 60 μg/mL of ox-LDL for 24 h. Cells in various groups were collected and suspended with 100 μL of binding buffer. PI and Annexin V-FITC solutions were then added to the cells. After incubation for 15 min, 400 μL of binding buffer was supplemented into the cells. The apoptosis rate of cells was assessed by using a flow cytometer.

### Intracellular ROS measurement

Intracellular ROS level was measured with 2,7-dichlorodihydrofluorescein diacetate (DCF-DA; Sigma-Aldrich, St. Louis, MO). After transfection with siRNAs and treatment with ox-LDL, HUVECs were incubated in a serum-free medium with 20 μM DCF-DA in an incubator for 30 min. The images were captured under a fluorescence microscope, or the cells were collected through trypsin digestion and then analyzed with a flow cytometer.

### SA-β-gal staining

Senescent HUVECs were detected using a SA-β-gal activity assay kit (Beyotime, Shanghai, China). HUVECs were seeded into a six-well culture plates. After transfection with siRNAs and treatment with ox-LDL, HUVECs were exposed to a fixing solution for 15 min. The plates were then incubated with a staining solution to detect the SA-β-gal activity in a humidified incubator without CO_2_ overnight. SA-β-gal-positive cells with a blue stain were captured under an optical microscope. The percentage of positive cells was calculated.

### Malondialdehyde (MDA) assay

The MDA content in the cells was detected using a MDA content detection kit (Solarbio Biotechnology, Beijing, China). Briefly, the treated HUVECs were harvested and resuspended in an extracting solution. The cell homogenates were then obtained by using an ultrasonic cell crusher (Scientz Biotechnology, Ningbo, China). After centrifugation at 8000 *g* for 10 min, the supernatants were mixed with the MDA reaction solution at 100 °C for 30 min and then centrifuged. The supernatants were put into a 96-well plate, and absorbance was measured at 532, 450, and 600 nm to detect the MDA content.

### Cell cycle assay

The HUVECs were seeded on six-well plates and cultured for 24 h. After transfection for 24 h, the cells were treated with 60 μg/mL of ox-LDL for 24 h. A cell cycle detection kit (KeyGen Biotech, Nanjing, China) was used to measure cell cycle distribution. Briefly, the cells were fixed with 70% ethanol, digested with 100 μL of RNase for 30 min, stained with 400 μL of propidium iodide, and were incubated for 30 min. Cell cycle distribution was detected and analyzed with a flow cytometer (BD Bioscience, San Jose, CA, USA).

### Western blotting

The HUVECs in each group were lysed using RIPA lysis buffer (Solarbio Biotechnology, Beijing, China). Equivalent amounts of proteins were separated with 12% SDS-polyacrylamide gels and then transblotted onto a PVDF membrane. The membranes were incubated with 5% dried nonfat milk buffer for 1 h to prevent nonspecific binding. They were then incubated with the relevant primary antibodies and with a secondary antibody. The protein bands in the membranes were detected by enhanced chemiluminescence detection reagents (Beyotime, Shanghai, China) and analyzed with Image J. Anti-Bcl-2, anti-Bax and anti-cleaved caspase-3 were from Cell Signaling Technology (Beverly, MA, USA). Anti-VCAM and anti-ICAM were from Abcam (Cambridge, MA, USA). Anti-p-IKBα, anti-IKBα, and anti-IL-8 were from Bioworld Technology (St. Louis Park, MN, USA).

### NF-κB nuclear translocation assay

The HUVECs were seeded on coverslips in 24-well culture plates and cultured for 24 h. After transfection for 24 h, the cells were treated with 60 μg/mL of ox-LDL for 24 h. A NF-κB activation-nuclear translocation assay kit (Beyotime, Shanghai, China) was used to detect the translocation of NF-κB from cytoplasm to nucleus. In brief, the cells were fixed with 4% paraformaldehyde for 5 minutes, followed by blocking buffer incubation for 1 h to prevent nonspecific binding. Then, cells were incubated with anti-NF-κB p65 at room temperature for 1 h, followed by incubation with Cy3-conjugated antibody at room temperature for 1 h. After incubation with DAPI for 5 min, the coverslips were sealed and observed under a fluorescence microscope.

### Histological examination

The aortic roots were excised and fixed overnight with 4% paraformaldehyde and then embedded in paraffin wax. The tissues were cut into 5 μm thickness. H&E staining and Masson staining were performed according to the instructions provided by the manufacturer (Solarbio Biotechnology, Beijing, China). The microscopic images of lesions in the aortic sinus were captured by an optical microscope. The percentage of lesion area and collagen area were analyzed using Image J.

### Oil Red O staining

Oil Red O staining was used to assess lipid accumulation in an atherosclerotic plaque. After the mice were euthanized, the whole thoracic aorta was isolated, cut lengthwise with scissors, placed in an Oil Red O solution for 15 min, and immersed in 70% ethanol until the normal tissues became white. The aorta was photographed, and Oil Red O-positive areas were analyzed using Image J.

### RNA-binding protein immunoprecipitation (RIP) assay

A magna RIP kit (Millipore, Bedford, MA, USA) was used to perform the RIP assay. In brief, the cell lysates were added to a magnetic bead conjugated with a human anti-SFPQ antibody (Sigma-Aldrich, St. Louis, MO) or control normal mouse IgG in RIP buffer. The immunoprecipitate was digested with proteinase K to purify the immunoprecipitated RNA. RT-qPCR was performed to verify the presence of NORAD.

### Serum lipid analyses

After 16 weeks of treatment, each group of mice fasted for 12 h. The serum was collected by removing their eyeballs. The concentrations of TC, TG, and LDL-C in the serum were determined with an automatic biochemical analyzer.

### Statistical analysis

Results were processed using SPSS 19.0. Data were shown as mean values ± standard deviation (SD). T test was used to analyze the data differences, and P < 0.05 indicated statistically significant differences.
